# DPP-4 Inhibition and the Path to Clinical Proof

**DOI:** 10.3389/fendo.2019.00376

**Published:** 2019-06-19

**Authors:** Bo Ahrén

**Affiliations:** Department of Clinical Sciences Lund, Lund University, Lund, Sweden

**Keywords:** type 2 diabetes, DPP-4, GLP-1, therapy, glucose-lowering, development

## Abstract

In the 1990s it was discovered that the enzyme dipeptidyl peptidase-4 (DPP-4) inactivates the incretin hormones glucagon-like peptide-1 (GLP-1) and glucose-dependent insulinotropic polypeptide (GIP). DPP-4 inhibition results in raised levels of the two incretin hormones which in turn result in lowering of circulating glucose through stimulation of insulin secretion and inhibition of glucagon secretion. Since then, several small orally available molecules have been developed with DPP-4 inhibitory action. Early studies in the 1990s showed that the DPP-4 inhibitors improve glycemia in animals. Subsequent clinical studies during the 2000s showed a glucose-lowering action of DPP-4 inhibitors also in human subjects with type 2 diabetes. This action was seen when DPP-4 inhibitors were used both as monotherapy and as add-on to other therapies, i.e., metformin, sulfonylureas, tiazolidinediones or exogenous insulin. The DPP-4 inhibitors were also found to have a low risk of adverse events, including hypoglycemia. Five of the DPP-4 inhibitors (sitagliptin, vildagliptin, alogliptin, saxagliptin and linagliptin) were approved by regulatory authorities and entered the market between 2006 and 2013. DPP-4 inhibitors have thereafter undergone long-term cardiovascular outcome trials, showing non-inferiority for risk of major acute cardiovascular endpoints. Also the risk of other potential adverse events is low in these long-term studies. DPP-4 inhibitors are at present included in guidelines as a glucose-lowering concept both as monotherapy and in combination therapies. This article summarizes the development of the DPP-4 inhibition concept from its early stages in the 1990s. The article underscores that the development has its basis in scientific studies on pathophysiology of type 2 diabetes and the importance of targeting the islet dysfunction, that the development has been made possible through academic science in collaboration with the research-oriented pharmaceutical industry, and that the development of a novel concept takes time and requires focused efforts, persistence and long-term perserverance.

## Introduction

Dipeptidyl peptidase-4 (DPP-4) inhibition is an established glucose-lowering therapy in type 2 diabetes. It has a low risk of hypoglycemia and other adverse events and is not associated with weight gain. It is used mainly as add-on to metformin when metformin alone is insufficient for glycemic control, particularly when there is a desire to minimize the risk for hypoglycemia. It is also used as first line therapy when metformin is not tolerated, in subjects with renal insufficiency and in combination with thiazolidinediones, sodium glucose transport protein 2 (SGLT2) inhibitors, and insulin. It may also be a possibility for DPP-4 inhibition as first line glucose-lowering therapy when an islet-directed approach is desirable.

The development of the DPP-4 inhibition concept for glucose-lowering therapy in type 2 diabetes originated on the fundament of the incretin concept. The term incretin was coined by Starling in the early 1900s to mean a gut hormone which stimulates the internal secretion of the pancreas (1). This concept was further developed by La Barre and Still (2) and Heller (3) in the 1930s when they showed that administration of gut extracts to experimental animals resulted in lowering of circulating glucose. They suggested that this effect is mediated by increased secretion of insulin. Furthermore, in the 1960s the novel radioimmunoassay technique made it possible to demonstrate that an oral administration of glucose indeed elicits a stronger increase in circulating insulin than an intravenous glucose administration even at the same glucose levels (4, 5). These important findings further documented the existence of the incretin concept in relation to oral glucose ingestion. The incretin concept was later developed further by identifying the gut hormones glucose-dependent insulinotropic polypeptide (GIP) and glucagon-like peptide-1 (GLP-1) as main incretin hormones (6, 7) and the development of GLP-1 as a potential treatment of type 2 diabetes (8). To leverage on this potential of GLP-1 as a glucose-lowering medication in T2D, DPP-4 inhibitors were developed to prevent the inactivation of endogenous GLP-1.

**Figure 1:**
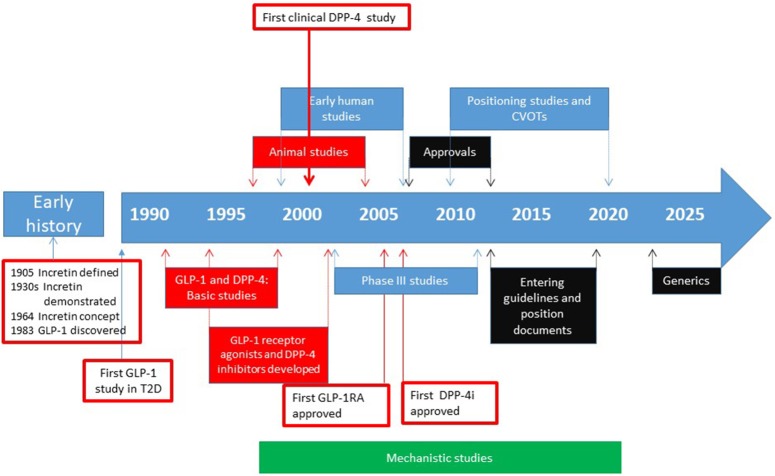
Approximate time line for the different phases in the path to clinical proof for DPP-4 inhibitors. The early history includes discovery and definition of the incretin concept and the discovery of GLP-1. The development of clinically therapy started with the idea that GLP-1 is a potential glucose-lowering agent. The development then went through establishing the structure and function, discovery of different DPP-4 inhibitors and their use in animal studies and early human studies. Then the phase III studies were performed followed by approval of DPP-4 inhibitors for use in patients with type 2 diabetes by FDA and EMA. Thereafter, post-approval positioning studies and cardiovascular outcome trials (CVOTs) were undertaken and the DPP-4 inhibitors entered guidelines and position documents. For future, generics will be introduced in the early 2020s which will reduce the cost of the therapy. In parallel with this development, mechanistic studies have been undertaken in both animals and humans, to establish the mechanism of action of DPP-4 inhibitors and effects of the compounds beyond their glucose-lowering action. Basic and experimental animal studies are shown in blue, human studies in red, regulatory phases in black and mechanistic studies (performed *in vitro*, in animals and in humans) shown in green. Arrows indicate approximate years for the various phases.

The development of DPP-4 inhibition as a glucose-lowering principle started in the 1990s with basic studies, continued in the 2000s with clinical studies for introduction of the concept to the market and was further developed in the 2010s with studies in special groups and long-term outcome studies with focus on cardiovascular diseases. Besides these clinical studies, also mechanistic studies have been performed to understand the underlying mechanisms of action and the low risk for hypoglycemia. Figure 1 shows the milestones of this development. This article will focus on the main steps in the path of developing DPP-4 inhibition as a glucose-lowering therapy.

## From Basic Studies to First Clinical Study

### GLP-1—A Novel Glucose-Lowering Agent During the 1990s

During the European Association for the Study of Diabetes (EASD) annual meeting in Copenhagen in 1990, the hypothesis was put forward that administration of the gut hormone GLP-1 may be a novel glucose-reducing therapy for both type 1 and type 2 diabetes (9). The data behind the hypothesis were drawn from a study in which GLP-1 had been infused intravenously to subjects with type 1 or type 2 diabetes during meal ingestion together with a variable insulin infusion to maintain glucose at euglycemic levels. The study demonstrated that the insulin requirement to maintain glycemia was markedly reduced by GLP-1, both in type 1 and type 2 diabetes. At the same time, circulating levels of glucagon were reduced. These data were published in 1992 (8). An accompanying editorial in the same issue of New England Journal of Medicine underlined the importance of the data for the vision of a novel incretin based therapy in diabetes (10).

Although this was the start of the development to use GLP-1 as a therapy in diabetes, GLP-1 had been discovered a few years earlier. It was thereby demonstrated that the proglucagon sequence contains the sequences of two peptides with structural similarities to glucagon. These two peptides were named GLP-1 and GLP-2. This finding was published in April, 1983, using the hamster as a model (11) and, in July, 1983, using the human proglucaon sequence (12). GLP-1 was subsequently localized to endocrine cells of the gut and early studies were initiated to establish its function. In 1987, GLP-1 was shown to stimulate insulin secretion in healthy humans, which showed that GLP-1 is an incretin hormone (7). GLP-1 was also early shown to inhibit glucagon secretion in animal studies (13, 14). Further studies showed that GLP-1 stimulates insulin secretion also in type 2 diabetes (15) and that overnight GLP-1 infusion reduces glucose levels in type 2 diabetes (16). An important 6-week study also demonstrated that continuous subcutaneous infusion of GLP-1 in subjects with type 2 diabetes reduces glycemia and body weight (17).

### Inhibition of GLP-1 Inactivation to Take Use of Its Glucose-Lowering Action

The challenge in the early 1990s of using GLP-1 as a glucose-reducing agent was its rapid inactivation which results in a short half-life of the peptide. Several attempts were undertaken to circumvent this, such as the formulation of a GLP-1 tablet for buccal administration, which was found to improve glycemia in type 2 diabetes (18). The breakthrough in the development was the demonstration that it is the enzyme DPP-4 that is responsible for the rapid inactivation of the hormone. This role of DPP-4 was first demonstrated in 1993 when human serum was incubated with GLP-1 in the absence or presence of the DPP-4 inhibitors lys-pyrrolidide or diprotin A, showing that the inactivation of GLP-1 was prevented by the DPP-4 inhibitors (19). This finding was later confirmed in a study from 1995, which used high pressure liquid chromatography in combination with radioimmunoassay making it possible to specifically analyze intact GLP-1 and its metabolites. This study demonstrated that the concentrations of intact GLP-1 increased and the formation of a GLP-1 metabolite was prevented in human plasma after incubation with DPP-4 inhibitors (20). These early *in vitro* studies formed the basis for the hypothesis that DPP-4 inhibition may be a potential novel therapeutic agent to stabilize endogenously released GLP-1. This potential initiated the search for DPP-4 inhibitors which were possible to use *in vivo* (21, 22). Similarly, it was also demonstrated that DPP-4 inactivates the other main incretin hormone GIP (19), which could further add to the beneficial effects of DPP-4 inhibition.

In parallel to the development of DPP-4 inhibition as a glucose-lowering concept, also DPP-4 resistant GLP-1 receptor agonists were developed. The first such to be approved for therapy was exenatide, which was approved by the US Food and Drug Administration (FDA) in 2005 (23). Later, several other GLP-1 receptor agonists have been approved (liraglutide, albiglutide, lixisenatide, dulaglutide and semaglutide) (24). The developmental path of GLP-1 receptor agonists is not covered in this article.

### Inhibiting the Proteolytic Activity of DPP-4 and Early Animal Studies

#### Inactivation of GLP-1 and GIP

DPP-4 is a catalytic glycoprotein which releases a dipeptide from oligopeptides by cleaving the peptides between the second and the third amino acids from the N-terminal end provided that the second amino acid is alanine or proline (25, 26). The two incretin hormones GLP-1 and GIP have both alanine as the second amino acid and therefore the two N-terminal amino acids of these peptides are released by DPP-4. These hormones are virtually inactive in stimulating insulin secretion after removal of the N-terminal di-peptide (27, 28). Therefore, the action of DPP-4 in reality means that the two incretin hormones are inactivated. The inactivation of GLP-1 and GIP by DPP-4 is the reason that the circulating half lifes of active (intact) GLP-1 and GIP are very short, since this degradation pathway is the main clearance pathway for GLP-1 and GIP.

#### Localization and Structure of DPP-4

DPP-4 was discovered as an enzyme already in 1966 (29). DPP-4 (or CD26 as it is also called) was later demonstrated to be expressed in several cell types, such as hepatocytes, glomerular cells, kidney tubular cells and endothelial cells as well as in islet endocrine cells (25, 26, 30, 31). DPP-4 is a protein which consists of 766 amino acids (32). It is attached to cell membranes with a short intracellular part (6 amino acids), a short transmembraneous part (22 amino acids) and a large extracellular part (738 amino acids) (33). Its catalytic site is located in a small five amino acid region localized toward the C-terminal end and centered around a serine positioned as amino acid number 630 (25, 34). On top of this, DPP-4 is a functional unit consisting of two identical DPP-4 proteins attached to each other forming a dimer. The organization of the dimer is such that the catalytic sites of the two DPP-4 molecules are located in close proximity to each other. Together the two catalytic sites form a pocket which has a high catalytic activity (35). The other parts of the DPP-4 molecule have other functions which are not related to enzymatic inactivation of GLP-1 and GIP, as for example immune function (36). DPP-4 also exists in a soluble form which circulates in plasma (36). This soluble form of DPP-4 does, however, not seem to be involved in glycemic regulation (37).

#### Development of DPP-4 Inhibitors

In the 1990s several stable, specific and orally active inhibitors of the catalytic site of DPP-4 were developed for the early *in vivo* studies. The developed DPP-4 inhibitors are small molecules which enter the catalytic pocket of the dimeric structure of DPP-4 and bind to the catalytic site which prevents the proteolytic activity. Some of the DPP-4 inhibitors are substrates of DPP-4 whereas others bind to the catalytic site without being degraded.

One of the earliest developed DPP-4 inhibitors was valine-pyrrolidide which functioned as a prototype for DPP-4 inhibition in early proof-of-concept experimental studies. Thus, it was shown in studies performed in 1995 in cynomolgus monkeys and rats that valine-pyrrolidide reduces glucose excursion after an oral glucose load (26). Furthermore, in anesthetized pigs, valine-pyrrolidide increased the concentration of intact GLP-1, prolonged the half-life of GLP-1 from 1 to 3 min and augmented the insulin response to intravenous GLP-1 administration (38). Moreover, valine pyrrolidide was also shown to increase the concentration of intact GIP, to prolong the half-life of GIP from 3 to 8 min and to potentiate the insulinotropic effect of GIP, resulting in enhanced glucose disappearance and a reduction in the glucose excursion after an intravenous glucose in anesthetized pigs (39). The effects of valine-pyrrolidide were also examined when given with a gastric glucose administration to high-fat fed glucose intolerant and control C57BL/6J mice (40). It was found that the increases in plasma GLP-1 and insulin after gastric glucose were potentiated by valine pyrrolidide and that the glucose tolerance was improved. In contrast, valine-pyrrolidide did not affect glucose-stimulated insulin secretion from isolated islets (40). This finding indicated that the effects of DPP-4 inhibition are mainly a consequence of its primary effect to prevent the inactivation of the incretin hormones.

Also other DPP-4 inhibitors were developed and examined during the 1990s. The DPP-4 inhibitor isoleucin-thiazolidide was found to augment insulin secretion and improve glucose tolerance in association with prolonging the half-life of GLP-1 in lean and obese Zucker rats (41). Furthermore, the DPP-4 inhibitor P32/98 was demonstrated to increase glucose tolerance and insulin secretion following daily administration during a 3 month study period in obese Zucker rats (42). In addition, two methanoprolinenitrile-containing dipeptide mimetics with DPP-4 inhibitory properties were found to reduce glucose levels in Zucker rats (43). These studies therefore formed firm evidence for the concept that DPP-4 inhibition may be beneficial in reducing glucose and the studies in animals formed a foundation for the proceeding to test the idea in humans.

### First Clinical Study With DPP-4 Inhibition

#### NVP DPP728 in Animals

**Figure 2:**
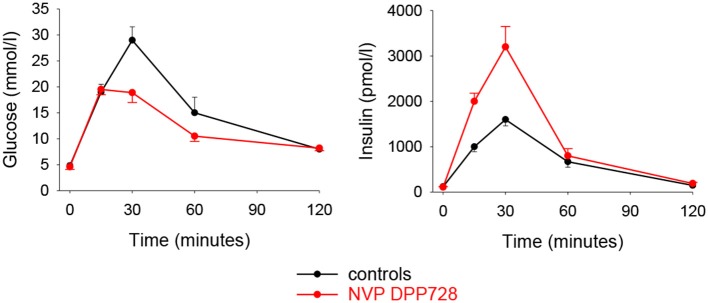
Plasma levels of glucose and insulin after administration of glucose (150 mg) through oral gavage to C57BL/6J mice which had been fed normal diet and given treatment with the DPP-4 inhibitor NVP DPP728 in the drinking water for 8 weeks. Controls were given drinking water without NVP DPP728. Means ± S.E.M are shown (*n* = 16 in each group). Area under the curve for glucose was significantly lower and area under the curve for insulin was significantly higher with NVP DPP728 than with controls. Data from Reimer et al. (47).

The first DPP-4 inhibitor which was used in a clinical study in type 2 diabetes was NVP DPP728. This inhibitor is chemically a cyanopyrrolidide compound which by itself is a substrate for DPP-4 with a slow dissociation rate which enables it to function as a competitive inhibitor of the enzyme (44). Initial experimental studies showed that NVP DPP728 improves glucose tolerance after oral glucose in cynomolgus monkeys (45). This inhibitor was also found to potentiate the insulin response to an oral glucose load in obese Zucker rats which resulted in improved glucose tolerance (46). Furthermore, long-term administration of this compound for 8 weeks in normal and high-fat fed mice improved glucose tolerance after gastric glucose gavage in association with increased plasma levels of insulin and intact GLP-1 (47). Figure 2 shows the results in these studies. A gastric gavage with 150 mg glucose increased circulating levels of glucose and insulin and 8 week pretreatment with NVP DPP728 reduced the increase in glucose and augmented the increase in insulin (47).

#### First Clinical Study

**Figure 3:**
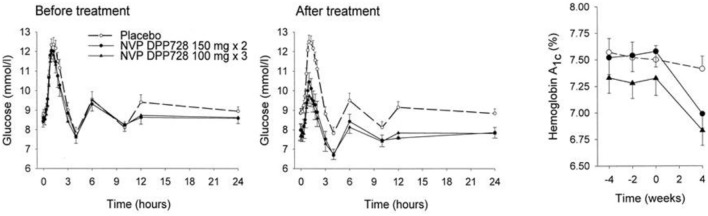
Twenty-four-hours glucose levels and HbA1c before and after 4 weeks of treatment with placebo (*n* = 32) or the DPP-4 inhibitor NVP DPP728 at 100 mg three times daily (*n* = 30) or 150 mg twice daily (*n* = 30) in subjects with drug-naïve type 2 diabetes. Means ± SEM are shown. NVP DPP728 significantly reduced glucose levels and HbA1c at both doses compared to placebo. Data from Ahrén et al. (49). Reprinted with permission from the American Diabetes Association.

The first clinical study with a DPP-4 inhibitor was performed with NVP DPP728 at five centers in Sweden and involved 93 patients with mild drug-naive type 2 diabetes (61 men and 32 women). Mean age was 64 years, mean BMI 27 kg/m^2^, mean HbA1c 7.4% (57 mmol/mol) and mean fasting glucose 8.5 mmol/l before the study. NVP DPP728 at the two doses of 100 mg x 3 or 150 mg x 2 or placebo was given for 4 weeks followed by a 24-hr study with standardized meals (total 2,000 kcal). It was found that NVP DPP728 at both doses reduced mean fasting glucose by ≈1.0 mmol/l, mean prandial glucose excursions by ≈1.2 mmol/l, and mean 24-h glucose levels by ≈1.0 mmol/l. Furthermore, insulin levels were sustained in spite of the reduced glucose levels. HbA1c was reduced to 6.9% (52 mmol/mol), i.e., by 0.5% (5 mmol/mol). There was no safety issue and tolerability was good. This first and conceptual important study on DPP-4 inhibition in subjects with type 2 diabetes was presented at the American Diabetes Association (ADA) meeting in Philadelphia in 2001 (48) and published the following year (49). Figure 3 shows the main data of this study: the reduction in 24-hr glucose levels after treatment with NVP DPP728 in relation to the unchanged level after placebo, and the reduction in HbA1c already after 4 weeks therapy.

This study represented a milestone since it ended the 10 year development of the concept of using DPP-4 inhibition for glucose-lowering action in type 2 diabetes with a clear proof-of-concept study. This phase in the path to clinical use of DPP-4 inhibitors was followed with a rapid development during the 2000s with clinical studies of several different DPP-4 inhibitors which ended in 2006–2013 with their approval for use in subjects with type 2 diabetes.

## Early Clinical Studies

Before the main development program was initiated for the DPP-4 inhibitors, several early clinical studies were undertaken, mainly with vildagliptin and sitagliptin. They underscored the potential of the concept and supported the initiation of large development programs.

### Vildagliptin

**Figure 4:**
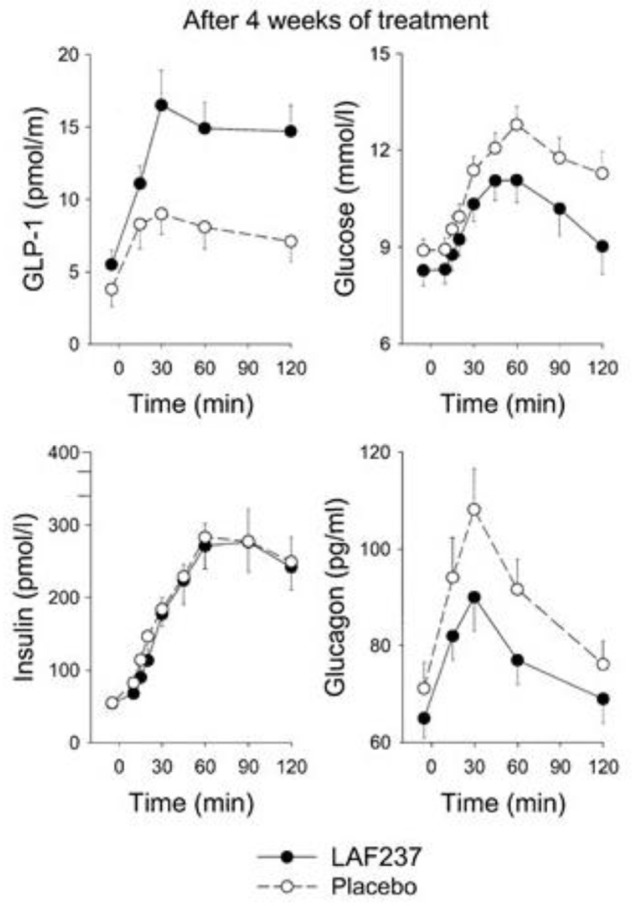
Intact (active) GLP-1, glucose, insulin, and glucagon levels after intake of breakfast (performed at time 0) after 4 weeks of treatment with placebo (*n* = 19) or the DPP-4 inhibitor vildagliptin (LAF237) at 100 mg daily (*n* = 18) in drug-naïve subjects with type 2 diabetes. Means ± sem are shown. Data from Ahrén et al. (52). Reprinted with permission from the American Diabetes Association.

The first clinical study with a DPP-4 inhibitor which eventually entered the market was presented in 2004. The DPP-4 inhibitor used was vildagliptin (LAF237) which is a competitive inhibitor and at the same time a substrate of DPP-4. It was developed chemically from NVP DPP728 and therefore also a cyanopyrrolidide compound (22, 50). After demonstrating its effect in animal studies, such as its effect to improve glucose tolerance after gastric glucose administration in mice (51), a 4 week clinical study was undertaken in drug-naïve patients with type 2 diabetes (52). The participants in this study had a mean age of 65 years, mean BMI of 27 kg/m^2^, mean fasting glucose of 9.0 mmol/l and mean HbA1c 7.1% (54 mmol/mol). Vildagliptin was found to reduce fasting glucose, 4-h prandial glucose excursion and mean 24-hr glucose by ≈1 mmol/l and HbA1c by 0.4% (5 mmol/mol) after 4 weeks. This was seen in association with increased baseline and post-prandial intact GLP-1 concentrations, reduced post-prandial glucagon levels and sustained insulin levels, which in the presence of lower glucose levels resulted in an increase of insulinogenic index as a sign of stimulated beta-cell function, which provided a first insight into the mechanism of action of DPP-4 inhibition in type 2 diabetes (Figure 4).

**Figure 5:**
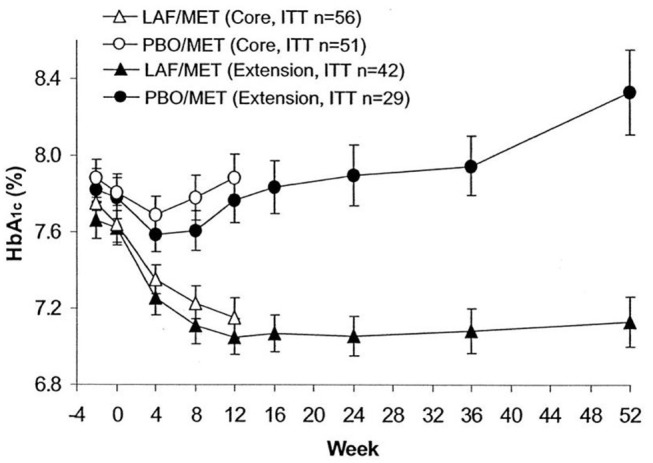
Time course of HbA_1c_ in a core study (open symbols) and an extension study (closed symbols) in 56 subjects with type 2 diabetes treated with the DPP-4 inhibitor vildagliptin (LAF237) and metformin and 51 subjects treated with placebo and metformin. Forty-two subjects with vildagliptin plus metformin and 29 subjects with placebo plus metformin participated in the extension. Data are means ± S.E.M. Data from Ahrén et al. (53). Reprinted with permission from the American Diabetes Association.

**Figure 6:**
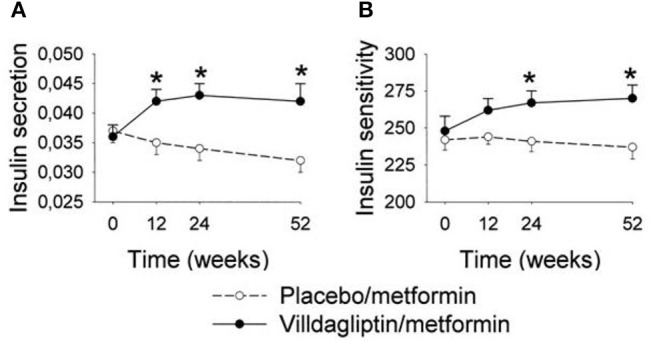
Insulin secretion (pmol/l 30 min)/(mmol/l) **(A)** and dynamic insulin sensitivity (oral glucose insulin sensitivity index, ml · min^−1^ · m^−2^) **(B)** at baseline (week 0) and after 12, 24, and 52 weeks of treatment with the DPP-4 inhibitor vildagliptin (*n* = 31) or placebo (*n* = 26) in metformin-treated subjects with type 2 diabetic. Means ± S.E.M. are shown. Data from Ahrén et al. (54) ^*^P < 0.05 between the groups. Reprinted with permission from the American Diabetes Association.

The initial study with vildagliptin in drug-naïve patients was followed by a 12 week study with a 40 week extension phase in 107 patients with metformin-treated type 2 diabetes (53). The study showed that vildagliptin reduced HbA1c from a mean baseline of 7.7% (61 mmol/mol) to 7.1% (54 mmol/mol) after 12 weeks. Thereafter, throughout the 40-week extension, HbA1c was stable during DPP-4 inhibition, whereas in placebo-treated patients, HbA1c increased throughout the study period, resulting in a difference in HbA1c between the groups after 52 weeks of 1.1% (11 mmol/mol) [Figure 5; (53)]. Furthermore, standardized meal tests were performed before and after the study period. The results of these tests revealed that vildagliptin stimulated beta-cell function and improved insulin resistance after 52 weeks [Figure 6; (54)].

### Sitagliptin

Another DPP-4 inhibitor which was developed early was sitagliptin (MK-431) (55). This is a selective beta-amino based competitive inhibitor of DPP-4. Sitagliptin was the first DPP-4 inhibitor that entered the market. A first study on sitagliptin as monotherapy was performed in 743 drug-naïve patients with type 2 diabetes using different doses for a treatment duration of 12 weeks (56). The patients had a mean baseline HbA1c of 7.7% (61 mmol/mol). After 12 weeks of treatment, sitagliptin had reduced HbA1c by 0.8% (8 mmol/mol) with a low risk for hypoglycemia and no weight gain. A second study examined sitagliptin as monotherapy for 18 weeks in drug-naïve patients (57). The study included 521 patients and HbA1c was reduced by sitagliptin from 7.7% (61 mmol/mol) to 7.1% (54 mmol/mol), again with low risk for hypoglycemia and no weight gain.

### Other DPP-4 Inhibitors

Three more inhibitors were developed at the time and went through development: saxagliptin [a cyanopyrrolidide compound; (58)], alogliptin [a pyromidinedione based compound; (59)], and linagliptin [a xanthine-based compound; (60)]. Table 1 shows the structure and other characteristics of these DPP-4 inhibitors. All DPP-4 inhibitors are orally active, specific for DPP-4 and with a high affinity to the enzyme, although their binding characteristics and pharmacokinetic properties differ (61).

**Table 1:** Structure, clinical dose (reflecting potency), and dates of approval by United States Food and Drug Administration (FDA) and European Medicines Agency (EMA) for the DPP-4 inhibitors, sitagliptin, vildagliptin, saxagliptin, alogliptin, and linagliptin, which were the first DPP-4 inhibitors reaching the market.

**Compound**	**Structure and chemical background**	**Clinical dose**	**Approval date by the FDA**	**Approval date by the EMA**
Sitagliptin	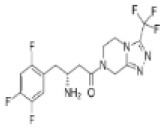	100 mg QD	October 17, 2006	March 20, 2007
	Beta-amino acid			
Vildagliptin	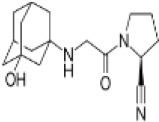	50 mg BID	−	September 25, 2007
	Cyanopyrrolidide			
Saxagliptin	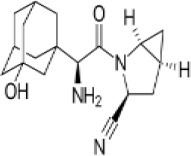	5 mg QD	July 31, 2009	September 30, 2009
	Cyanopyrrolidide			
Alogliptin	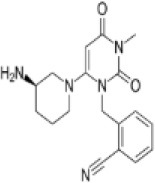	25 mg QD	May 2, 2011	August 23, 2011
	Pyrimidinedione			
Linagliptin	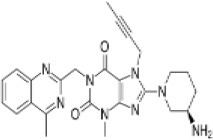	5 mg QD	January 25, 2013	September 18, 2013
	Xanthine			

### Mechanistic Evidence of Pathophysiologic Target

Besides the development of various efficient DPP-4 inhibitors and the demonstration that they have glucose-lowering properties, an important factor behind the concept of DPP-4 inhibition was that it targets the basic pathophysiological processes in type 2 diabetes. This concept evolved during the 1990s and 2000s when mechanistic studies were undertaken in parallel to the path of DPP-4 inhibition to clinical studies. DPP-4 inhibitors were shown to prevent the inactivation of GLP-1 and GIP resulting in a stimulation of insulin secretion and inhibition of glucagon secretion in a glucose-dependent manner (22, 26, 52, 54, 62). In particular the stimulation of beta cell function and inhibition of glucagon secretion were important, since insufficient insulin secretion and hypersecretion of glucagon were at that time emerging as the key defects underlying type 2 diabetes (63, 64). Islet dysfunction was also shown to exist already before the onset of type 2 diabetes in subjects with impaired glucose tolerance, i.e., at risk for developing diabetes (65). These findings suggested that DPP-4 inhibition targets the early causes of initiation of type 2 diabetes and the concept emerged that the treatment may be used as an early therapy during diabetes development (62). This idea initiated in turn several studies with different DPP-4 inhibitors in drug-naïve patients and in metformin-treated patients, i.e., patients in the early stage of the disease.

## Key Development Studies

The next step in the path of DPP-4 inhibition to approval for clinical use was the development programs for the DPP-4 inhibitors sitagliptin, vildagliptin, saxagliptin, alogliptin, and linagliptin. In these phase III studies the DPP-4 inhibitors were examined as monotherapy in drug-naïve patients and as add-on to on-going therapy with metformin, sulfonylurea therapy, thiazolidinedione therapy and therapy with exogenous insulin in type 2 diabetes (Table 2). Results on glycemic efficacy of these crucial studies are summarized here for the doses which were selected as the clinical doses.

**Table 2:** Development studies with five DPP-4 inhibitors: number of examined subjects and placebo-adjusted change in HA1c when DPP-4 inhibitors were added to drug-naïve subjects with type 2 diabetes or as add-on to metformin, sulfonylurea, thiazolidinediones, or insulin in placebo-controlled 24 or 26 week studies.

**DPP-4 inhibitor**	**Variable**	**Treatment to which DPP-4 inhibition was added**				
		**Drug-naive**	**Metformin**	**Sulfonylurea**	**Thiazolidinedione**	**Insulin**
Sitagliptin	No of subjects	741	701	441	353	641
	ΔHbA1c (%)	0.8	0.7	0.7	0.7	0.6
	(mmol/mol)	8	7	8	8	6
	Reference	66	71	78	84	89
Vildagliptin	No of subjects	354	544	515	463	449
	ΔHbA1c (%)	0.7	1.1	0.7	1.0	0.7
	(mmol/mol)	7	11	7	10	7
	Reference	67	72	79	85	90
Saxagliptin	No of subjects	401	743	768	565	455
	ΔHbA1c (%)	0.5	0.8	0.7	0.7	0.4
	(mmol/mol)	5	8	7	7	4
	Reference	68	73	80	86	91
Alogliptin	No of subjects	329	527	500	493	390
	ΔHbA1c (%)	0.5	0.5	0.5	0.8	0.6
	(mmol/mol)	5	5	5	8	6
	Reference	69	74	81	87	92
Linagliptin	No of subjects	503	701	1,058	272	1,261
	ΔHbA1c (%)	0.7	0.6	0.6	0.6	0.7
	(mmol/mol)	7	6	6	6	7
	Reference	70	75	82	88	93

*Study populations, study design and baseline HbA1c varied between the studies. Reported number of subjects are numbers for the entire studies. All studies had at least two arms (study medication and placebo). Change in HbA1c shown for clinically relevant dose*.

### Placebo-Controlled Studies With DPP-4 Inhibition as Monotherapy

**Figure 7:**
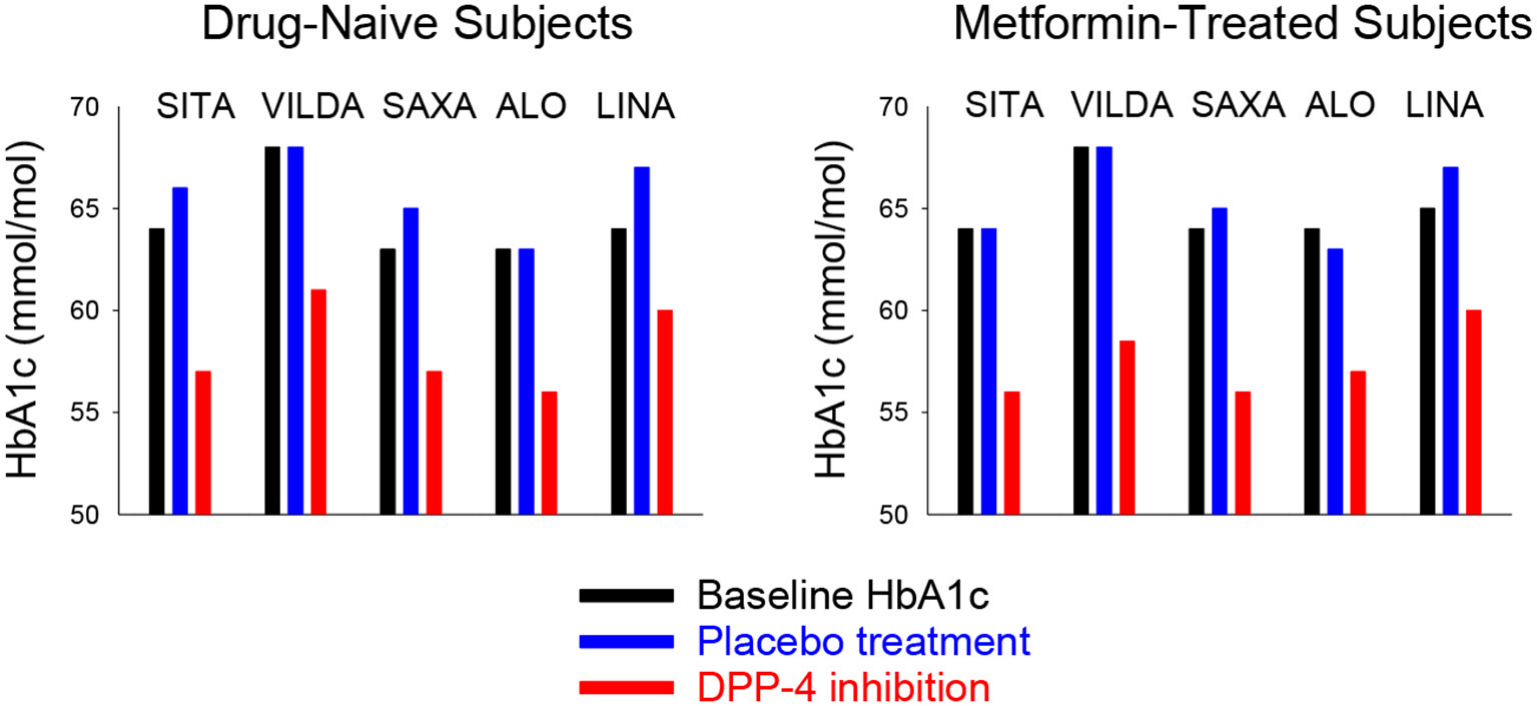
Mean baseline HbA1c and mean HbA1c after 24–26 weeks of treatment with placebo or a DPP-4 inhibitor in drug-naïve (**Left**) or metformin-treated (**Right**) subjects with type 2 diabetes in studies reported in Table 2. SITA, sitagliptin; VILDA, villdaglipin; SAXA, saxagliptin; ALO, alogliptin; LINA, linagliptin.

In drug-naïve subjects with type 2 diabetes who were treated with diet and life style, the DPP-4 inhibitors were examined as monotherapy in placebo-controlled 24–26 week studies with a total number of 2,328 subjects [Table 2; (66–70)]. Baseline HbA1c in these studies was 7.9–8.4% (63–68 mmol/mol). Figure 7 shows the effects of DPP-4 inhibitors and placebo in these studies. The DPP-4 inhibitors reduced HbA1c by 0.5–0.8% (5–8 mmol/mol) after 24–26 weeks. Body weight did not change significantly and all DPP-4 inhibitors were well-tolerated. There was no difference in the (low) numbers of confirmed hypoglycemia between DPP-4 inhibition and placebo and the occurrence of adverse events did not differ between the study groups except a slight increase in gastrointestinal adverse events in one of the studies (66).

### Placebo-Controlled Studies With DPP-4 Inhibition as Add-on to Ongoing Metformin Therapy

The DPP-4 inhibitors were also examined as add-on to on-going metformin in subjects with type 2 diabetes in 24–26 week studies with a total number of 3,216 subjects [Table 2; (71–75)]. Baseline HbA1c in these studies was 7.9–8.4% (63–68 mmol/mol). Figure 7 shows the effects of DPP-4 inhibitors and placebo in these studies. The placebo-adjusted reduction in HbA1c was 0.5–1.1% (5–11 mmol/mol). The DPP-4 inhibitors were well-tolerated, body weight was not significantly different from the placebo groups and there was no difference between the groups in the (low) numbers of confirmed hypoglycemia. Similarly, the occurrence of adverse events did not differ between the study groups.

**Figure 8:**
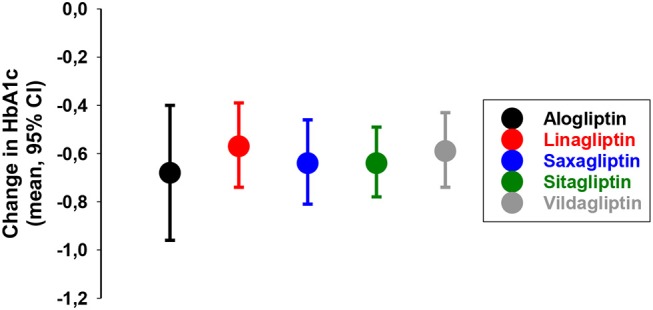
Placebo-adjusted change in HbA1c from 83 randomized clinical trials when a DPP-4 inhibitor was added to on-going therapy with metformin in subjects with type 2 diabetes as reported in a meta-analysis (76).

The add-on of DPP-4 inhibition to metformin therapy has also been the subjects of several other studies. In 2014, a meta-analysis of 83 randomized clinical trials were performed showing that DPP-4 inhibition reduced HbA1c by ≈0.4–0.5% (4–5 mmol/mol) when added to metformin [Figure 8, (76)]. Therefore, the combination of DPP-4 inhibition with metformin was already early suggested as a clinical important combination (77).

### Placebo-Controlled Studies With DPP-4 Inhibition as Add-on to Ongoing Sulfonylurea Therapy

The DPP-4 inhibitors were as well-examined when added to on-going sulfonylurea in subjects with type 2 diabetes in 24–26 week placebo-controlled studies with a total number of 3,282 subjects [Table 2; (78–82)]. The studies had slightly different designs. Four of the studies had constant sulfonylurea dosage throughout the study (78, 79, 81, 82), whereas one study added the DPP-4 inhibitor to submaximal dose of sulfonylurea and compared this combination with up-titration of the sulfonylurea (80). In three of the studies, the sulfonylurea was used in monotherapy (79–81), in one study the sulfonylurea was used in combination with metformin (82) and one study allowed sulfonylurea both as monotherapy and in combination with metformin (78). Furthermore, in two studies, glibenclamide was used as the sulfonylurea (78, 79), in two studies the sulfonylurea was glibenclamide (glyburide) (80, 81) and in one study different sulfonylureas were used (82). Despite these differences, the outcomes were fairly similar. Baseline HbA1c in these studies varied from 8.1 to 8.5% (65–69 mmol/mol). The placebo-adjusted reduction in HbA1c by the DPP-4 inhibitors varied from 0.5 to 0.7% (5–8 mmol/mol). The DPP-4 inhibitors were well-tolerated and body weight was not significantly different between DPP-4 inhibition and placebo. In four of the studies there was a slightly increased rate of hypoglycemia episodes in the combination with DPP-4 inhibitor and sulfonylura compared to sulfonylurea alone (78, 79, 81, 82). This increased risk for hypoglycemia with this combination has later been confirmed in other studies and a meta-analysis showed an approximately 50% increased risk for hypoglycemia when DPP-4 inhibitors are combined with sulfonylurea (83). Therefore, a clinical advice is that the dose of sulfonylurea should be reduced when combined with DPP-4 inhibitors.

### Placebo-Controlled Studies With DPP-4 Inhibition as Add-on to Ongoing Thiazolidinedione Therapy

When added to on-going therapy with a thiazolidinedione in subjects with type 2 diabetes, the DPP-4 inhibitors were studied in 24–26 week placebo-controlled studies with a total number of 2,146 subjects [Table 2; (84–88)]. The studies had different designs. In two studies, pioglitazone was used as monotherapy (84, 85), one study allowed rosiglitazone or pioglitazone as monotherapy (86), in one study pioglitazone was used alone or with metformin and/or sulfonylurea (87) and one study used pioglitazone with metformin (88). Baseline HbA1c in these studies varied from 8.1 to 8.7% (65–72 mmol/mol). The placebo-adjusted reduction in HbA1c by DPP-4 inhibitors varied between 0.6 and 1.0% (6–10 mmol/mol). The DPP-4 inhibitors were well-tolerated in the combination with the thiazolidinedione, body weight was not significantly different between DPP-4 inhibition and placebo and there was no difference in rates of hypoglycemia between the groups.

### Placebo-Controlled Studies With DPP-4 Inhibition as Add-on to Ongoing Exogenous Insulin Therapy

Sitagliptin, vildagliptin, saxagliptin, alogliptin, and linagliptin were also examined when added to on-going therapy with insulin in subjects with type 2 diabetes in 24–26 week placebo-controlled studies with a total number of 3,196 subjects [Table 2; (89–93)]. Study populations were treated either with stable dose of insulin with or without metformin (89–92) or, in one study, stable dose of insulin with or without metformin or pioglitazone (93). Baseline HbA1c in these studies varied from 8.3 to 9.3% (67–78 mmol/mol). The DPP-4 inhibitors reduced HbA1c vs. placebo. The placebo-adjusted HbA1c reduction varied between 0.4 and 0.7% (4–7 mmol/mol). The DPP-4 inhibitors were well-tolerated in combination with insulin and did not cause weight gain. There was no difference in rate of hypoglycemia between DPP-4 inhibition and placebo in four of the studies (90–93) whereas one study showed an increased rate of hypoglycemia with the combination (81).

## Approval and Therapeutic Guidelines

### Approval

The next step after these development studies was the approval of DPP-4 inhibitors by regulatory bodies. Between 2006 and 2013, the DPP-4 inhibitors were approved for glucose-lowering use in type 2 diabetes both by the FDA and by the European Medicines Agency (EMA). Table 3 shows the approval dates for the five DPP-4 inhibitors.

**Table 3:** Cardiovascular outcomes trials with DPP-4 inhibitors, name, number of subjects, and duration.

**DPP-4 inhibitor**	**Name of trial**	**Number of subjects**	**Median follow up period (year)**	Hazard ratio for primary endpoint (95% CI^*^)	**References**
Saxagliptin	SAVOR-TIMI	16,492	2.1	1.00 (0.89; 1.12)	(94)
Alogliptin	EXAMINE	5,280	1.5	0.96 (0.76; 1.16)	(95)
Sitagliptin	TECOS	14,671	3.0	0.98 (0.88; 1.09)	(96)
Linagliptin	CARMELINA	6,979	2.2	1.02 (0.89; 1.17)	(97)
Linagliptin	CAROLINA	6,033^**^	6.0^**^	Not reported	(98)

* *CI, confidence interval*.

** *These results reported in a press release from Boehringer Ingelheim, February 19, 2019*.

*Primary endpoint: composite of cardiovascular death, myocardial infarction, or ischemic stroke (94), composite of death from cardiovascular causes, non-fatal myocardial infarction, or non-fatal stroke (95), composite of cardiovascular death, non-fatal myocardial infarction, non-fatal stroke, or hospitalization for unstable angina (96), or time to first occurrence of the composite of cardiovascular death, non-fatal myocardial infarction, or non-fatal stroke (97), respectively*.

### DPP-4 Inhibitors in Other Countries

In parallel with this development, other DPP-4 inhibitors were developed in Japan and South Korea. These DPP-4 inhibitors are anagliptin, evogliptin, gemigliptin, omarigliptin, teneligliptin, trelagliptin, gasogliptin, evogliptin, and retagliptin (99).

### Positioning Discussions

Following the approval of the DPP-4 inhibitors, their positioning in the therapy was discussed in the scientific and clinical community as well as among regulatory and health care system bodies. DPP-4 inhibitors were thereby considered mainly as early therapy in combination with metformin in subjects with type 2 diabetes. However, due to the higher cost for the DPP-4 inhibitors, compared to sulfonylureas and thiazolidinediones, and the lack of long-term studies, it took several years for agencies responsible for guidelines to place DPP-4 inhibitors as a suggested glucose-lowering agent. An early recognition of DPP-4 inhibitors as glucose-lowering agents was the joint position document from EASD and ADA in 2012 (100). It suggested metformin as the first-line glucose-lowering pharmacotherapy and when metformin alone is insufficient for achieving glycemic control, any of sulfonylureas, thiazolidindediones, DPP-4 inhibitors, GLP-1 receptor agonists, or insulin could be added; these could then be added in combination also as triple therapy. The position document summarized that the efficacy of DPP-4 inhibitors to lower glucose is intermediate when compared to other therapies, that DPP-4 inhibitors are neutral in relation to change in body weight, that major side effects are rarely seen for DPP-4 inhibitors, that the risk for hypoglycemia is low and that the cost is high. These characteristics were enforced in the revision of the position document in 2015, when also SGLT2 inhibitors were added as a possible agent to add-on to metformin (101).

## Comparison With Other Therapies and Studies in Renal Impairment

To judge the positioning of the therapy, a further step in the development of DPP-4 inhibition was comparisons with other therapies. Also studies on efficacy and safety in subgroup of patients of interest, mainly in subjects with renal insufficiency were important in this phase of development.

### Comparison With Sulfonylurea

**Figure 9:**
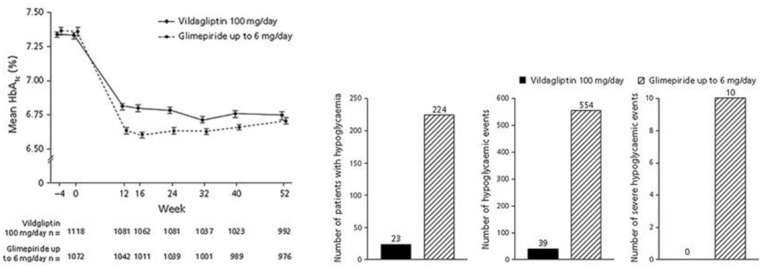
Mean HbA_1c_, number of patients with hypoglycemia, number of hypoglycemic events and number of severe hypoglycemia events during treatment with the DPP-4 inhibitor vildagliptin plus metformin or the sulfonylurea glimepiride plus metformin in subjects with type 2 diabetes. Means ± S.E.M. are shown. Data from Ferrannini et al. (103). Reprinted with permission from Wiley.

Studies comparing DPP-4 inhibitors with sulfonylurea compared sitagliptin with glipizide (102), vildagliptin with glimepiride (103), saxagliptin with glipizide (104), alogliptin with glipizide (105), and linagliptin with glimepiride (93). The studies were of long-term duration and lasted for 1 year (102–105) or 2 years (106). The total number of subjects in these studies was 6,037. Baseline HbA1c was 7.3–7.7% (56–61 mmol/mol). The studies had similar outcomes: DPP-4 inhibition and sulfonylurea both reduced HbA1c, DPP-4 inhibition had no weight gain, which on the contrary was seen with sulfonylurea, and there was a marked difference in hypoglycemia episodes. Figure 9 shows results in one of the studies (103). In this study, subjects on metformin monotherapy with mean baseline HbA1c 7.3% (56 mmol/mol) received vildagliptin or glimepiride (titrated up to 6 mg/day; mean dose 4.5 mg/day). After 52 weeks, HbA1c was reduced by 0.4% (4 mmol/mol) by vildagliptin and 0.5% (5 mmol/mol) by glimepiride, body weight was slightly reduced by vildagliptin (0.2 kg) but increased by glimepiride (1.89 kg) and there was a 10-fold higher incidence of hypoglycemia in the glimepiride group. Therefore, these studies showed advantage of DPP-4 inhibition over sulfonylureas as add-on to metformin in regard both to lower risk of hypoglycemia and no weight gain.

### Comparison With Thiazolidinediones

Studies comparing vildagliptin with rosiglitazone as monotherapy (107), vildagliptin with pioglitazone as add-on to metformin (108), and sitagliptin with pioglitazone as monotherapy (109) have been undertaken. The studies have shown that the reduction in HbA1c was not different between DPP-4 inhibitors and thiazolidinediones, that adverse events, including rate of hypoglycemia, did not differ between the groups and that body weight was increased by thiazolidinedione therapy but not changed by DPP-4 inhibition.

### Comparison With GLP-1 Receptor Agonists

Studies have compared sitagliptin with GLP-1 receptor agonists in drug naïve subjects with type 2 diabetes [exenatide, (110)] or as add on to other therapy for exenatide (111), semaglutide (112), albiglutide (113), and dulaglutide (114). These studies showed a lower reduction in HbA1c by sitagliptin than by GLP-1 receptor agonists. Body weight was in general reduced by GLP-1 receptor agonists but not by sitagliptin, and adverse events were higher for GLP-1 receptor agonists, being mainly gastrointestinal.

### Comparison With SGLT2 Inhibitors

Studies comparing sitagliptin with ertugliflozin (115), canagliflozin (116), or empagliflozin (117) have been undertaken. Results showed that sitagliptin is equal to SGLT2 inhibition (115) or that SGLT2 inhibition is more efficacious (116, 117) in reducing HbA1c. Furthermore, risk of hypoglycemia was equal between DPP-4 inhibition vs. SGLT2 inhibition (115, 117) or higher for SGLT2 inhibition (116), whereas all studies showed that body weight is reduced by SGLT2 inhibitors but not by sitagliptin and adverse events, such as genital infections, are higher for SGLT-2 inhibitors than for sitagliptin.

### Renal Insufficiency

DPP-4 inhibitors have also been examined in placebo-controlled studies in subjects with type 2 diabetes and chronic renal insufficiency, including patients with end-stage renal disease on dialysis (118–121). The results show that DPP-4 inhibition reduces HbA1c in these patients and that the treatment is well-tolerated with similar rate of adverse events as placebo. DPP-4 inhibition may therefore be used also in subjects with renal insufficiency.

## Status After the Development Studies

### Glycemic Effect

To summarize the studies undertaken with DPP-4 inhibitors as monotherapy or in combination with other glucose-lowering therapies in the years around 2010, they showed that the inhibitors reduce HbA1c by ≈0.5–0.8% (5–8 mmol/mol), have a low risk for hypoglycemia (except in combination with sulfonylurea), and are weight neutral.

### Other Effects

Although not significant in all studies, *post-hoc* analysis of the placebo-controlled studies in phase III showed that DPP-4 inhibitors slightly lower blood pressure, improve post-prandial and fasting lipemia, reduce inflammatory markers, diminish oxidative stress, improve endothelial function, and reduce platelet aggregation in type 2 diabetes (122).

### Adverse Events

An overall experience in studies with DPP-4 inhibitors was that they have a low risk of adverse events, including hypoglycemia. Adverse events, such as infections, headache, gastrointestinal adverse events, and skin lesions were reported in some studies. However, when summarizing studies together, including studies undertaken throughout the first 10 years after approval, the DPP-4 inhibitors had no clear signal of an adverse event and are therefore considered safe (123).

A special discussion has been undertaken in relation to a risk for acute pancreatitis and other pancreatic disease, since an increased risk of these diseases have been observed during incretin therapy in some studies (124, 125). A mechanism for pancreatitis during DPP-4 inhibition may be the chronic stimulation of pancreatic acinar and duct cells by GLP-1, since these cells express GLP-1 receptors and proliferate in response to chronic stimulation by GLP-1 in experimental studies (126). An increased risk was also supported by results of a large meta-analysis comprising a total of 36 double-blind randomized clinical trials with DPP-4 inhibitors with a total of 54,664 patients: there was a 58% increased risk of acute pancreatitis with DPP-4 inhibitors compared with other therapies (127). In contrast, however, other studies have shown no significantly increased risk for acute pancreatitis with incretin therapies compared with other glucose-lowering therapies (128). Similarly, a real-world analysis of 225,898 patients showed that the risk for pancreatitis with DPP-4 inhibitors is not higher as with other glucose-lowering drugs (129). Because of the controversy of this important question, the FDA and EMA jointly evaluated all studies and concluded that there was no evidence of a causal association between incretin therapy and pancreatic adverse events (130). Nevertheless, cautious should be undertaken and further evaluation is warranted. Also, the potential risk of pancreatitis has been added to the label of DPP-4 inhibitors.

## Cardiovascular Outcome Trials

The path for DPP-4 inhibition during the 2010s was centered around the several cardiovascular outcome trials performed with DPP-4 inhibitors. Between 2013 and 2019, five of the studies were published or reported (Table 3). The studies were all performed in subjects at risk for cardiovascular diseases and were initially designed and powered for cardiovascular safety. However, the results are also important for documenting the long-term general safety of the DPP-4 inhibitors and the discussions of a potential beneficial cardiovascular effect of glucose-lowering therapies.

### The Saxagliptin CVOT

In the Saxagliptin Assessment of Vascular Outcomes Recorded in Patients with Diabetes Mellitus (SAVOR)–Thrombolysis in Myocardial Infarction (TIMI) (SAVOR-TIMI) study, 16,492 patients with type 2 diabetes who either had a previous history of cardiovascular disease or were at risk for such events were randomized to treatment with saxagliptin or placebo for a median follow-up period of 2.1 years. The primary end point (a composite of cardiovascular death, myocardial infarction, or ischemic stroke) occurred in 613 patients in the saxagliptin group and in 609 patients in the placebo group (7.3 and 7.2%), which was not significantly different. Adverse events, including pancreatitis, were not different between the groups, except that more patients in the saxagliptin group (3.5%) than in the placebo group (2.8%) were hospitalized for heart failure (94).

### The Alogliptin CVOT

In the Examination of Cardiovascular Outcomes with Alogliptin vs. Standard of Care (EXAMINE) study, 5,280 patients with type 2 diabetes and either an acute myocardial infarction or unstable angina requiring hospitalization within the previous 15–90 days were randomized to alogliptin or placebo in addition to existing glucose-lowering and cardiovascular drug therapy for a median follow-up of 1.5 years. The primary end point (a composite of death from cardiovascular causes, non-fatal myocardial infarction, or non-fatal stroke) occurred in 305 patients with alogliptin (11.3%) and in 316 patients with placebo (11.8%), which was not significantly different. Adverse events, such as hypoglycemia, cancer, pancreatitis, and heart failure were similar with alogliptin and placebo (95).

### The Sitagliptin CVOT

In the Trial Evaluating Cardiovascular Outcomes with Sitagliptin (TECOS) study, 14,671 patients with type 2 diabetes and established cardiovascular disease were randomized to addition of sitagliptin or placebo to existing therapy for a median follow-up period of 3.0 years. The primary outcome (a composite of cardiovascular death, non-fatal myocardial infarction, non-fatal stroke, or hospitalization for unstable angina) occurred in 839 patients in the sitagliptin group (11.4%) and 851 patients in the placebo group (11.6%), which was not significantly different. Furthermore, rates of hospitalization for heart failure and acute pancreatitis did not differ between the groups (96).

### The Linagliptin CVOTs

In the Cardiovascular and Renal Microvascular Outcome Study With Linagliptin (CARMELINA), 6,979 patients with type 2 diabetes and high risk of cardiovascular and kidney events were randomized to linagliptin och placebo for a median follow-up of 2.2 years. The primary outcome (time to first occurrence of the composite of cardiovascular death, non-fatal myocardial infarction, or non-fatal stroke) occurred in 12.4 and 12.1% of subjects in the linagliptin and placebo groups, respectively, which was not significantly different between the groups. There was no significant difference in adverse events, including hypoglycemia, and hospitalization for heart failure, between the groups (97).

In the Cardiovascular Outcome study of Linagliptin vs. Glimepiride (CAROLINA) study, the cardiovascular safety of linagliptin was compared with the sulfonylurea glimepiride on top of standard of care in 6,033 subjects with type 2 diabetes and increased cardiovascular risk or established cardiovascular disease over a median follow-up period of 6 years. The results showed that the primary outcome (time to first occurrence of cardiovascular death, non-fatal myocardial infarction, or non-fatal stroke) did not differ significantly between the groups^1^.

### Summary of CVOT With DPP-4 Inhibitors

These studies therefore show that DPP-4 inhibitors are safe from a cardiovascular point of view in subjects at risk for cardiovascular diseases and type 2 diabetes and, also, that risk for other adverse events, such as hypoglycemia or pancreatitis, is low. Furthermore, the increased rate of hospitalization for heart failure, which was observed in one of the studies (122) was not confirmed in the other studies (95–97). Overall, therefore, these studies assure the safety of DPP-4 inhibitors when used as glucose-lowering treatment of type 2 diabetes. On the other hand, there was no superiority with DPP-4 inhibitors vs. the placebo group in major acute cardiovascular events, as has recently been shown for cardiovascular outcome trials with the GLP-1 receptor agonists liraglutide (131), semaglutide (132), albiglutide (133) and dulaglutide^2^ and the SGLT2 inhibitors empagliflozin (134) and canagliflozin (135).

### Importance of Long-Term Evaluation of Adverse Events

The results on the long-term safety are important since it was initially a concern that DPP-4 inhibition may be associated with adverse events. This concern was related to the action of DPP-4 to cleave a number of bioactive peptides with alanine or proline as the second amino acid from the N-terminal end apart from GLP-1 and GIP, such as neuropeptide Y, gastrin-releasing peptide, substance P, and various chemokines (25, 26). Since these biologically active peptides are inactivated also by other pathways, the DPP-4 action is not as dependent for their inactivation as it is for GLP-1 and GIP. Therefore, it was not surprising that the risk for adverse events with DPP-4 inhibitors is not different from the risk in placebo groups. Nevertheless, even longer follow-up periods are required for safety studies on the compounds.

## Low Risk of Hypoglycemia

A consisting finding with DPP-4 inhibition has been its low risk for hypoglycemia. This low risk of hypoglycemia with DPP-4 inhibition is also underlined in guidelines and position documents (100, 101). This is also evident from the placebo-controlled trials in which the risk of hypoglycemia in general has been found not to be different between DPP-4 inhibition and placebo (66–97). There are several reasons for this low risk of hypoglycemia with DPP-4 inhibitors. One reason is the glucose-dependency of the action of GLP-1 (64). A certain glucose level is required for GLP-1 to stimulate insulin secretion and inhibit glucagon secretion and if glucose levels drop below this, these effects vanish which lowers the risk of further reduction in glucose levels. Another mechanism may be achieved through the action of GIP, the level of which is increased during DPP-4 inhibition. Thus, GIP is known to stimulate glucagon secretion during hypoglycemia (136). This effect is important since the glucagon counter-regulation to hypoglycemia is important to restore glucose during hypoglycemia. When GIP levels therefore are elevated, as during DPP-4 inhibition, the glucagon counter-regulation to hypoglycemia may be supported by the action of GIP. This mechanism is also supported by animal studies which have suggested the existence of a GIP-glucagon axis which is of particular importance during hypoglycemia (137).

**Figure 10:**
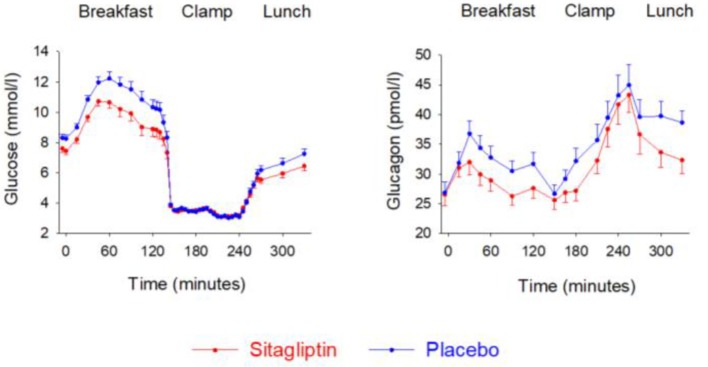
Plasma levels of glucose and glucagon during breakfast, insulin-induced hypoglycemia and subsequent lunch in 28 elderly (65–84 years) subjects with metformin-treated type 2 diabetes after 4 weeks of add-on therapy with sitagliptin or placebo. Means ± S.E.M. are shown. Data from Farngren et al. (140). Reprinted with permission from Wiley.

Whether glucagon counter-regulation to hypoglycemia is affected during DPP-4 inhibition in type 2 diabetes has been studied in drug-naïve subjects (138), in subjects when a DPP-4 inhibition was added on to insulin therapy (139), and in elderly subjects with metformin-treated type 2 diabetes (140). These studies have used the hyperinsulinemic, hypoglycemic clamp technique and have determined glucagon counter regulation. Results showed that glucagon counter-regulation during hypoglycemia is sustained during DPP-4 inhibition. Figure 10 shows results from the study in elderly subjects (140). The study examined the counter-regulation to hypoglycaemia in 28 elderly subjects (65–84 years) with metformin-treated type 2 diabetes after addition of sitagliptin or placebo for 4 weeks in a cross-over design. A two-step hyperinsulinemic hypoglycemic clamp (3.5 and 3.1 mmol/l glucose) was undertaken after each treatment period. It was found that the glucagon response at 3.5 mmol/l was lower after sitagliptin than after placebo whereas at 3.1 mmol/l, glucagon did not differ between the two. The study therefore shows that during hypoglycaemia, glucagon counter-regulation is sustained during DPP-4 inhibition also in the elderly and that there seems to be a glucose threshold between 3.1 and 3.5 mmol/l for the relieve of the inhibition by DPP-4 inhibition of glucagon.

## Current Status and Future Outlook

### Current Recommendations

Following the recent demonstration of several long-term cardiovascular outcomes trials and other new studies, the EASD/ADA position statement on management of hyperglycemia in type 2 diabetes was revised again in 2018 (141). The suggestion in this novel position document is that metformin is still the first line glucose-lowering pharmacotherapy. When this is insufficient for managing the hyperglycemia, GLP-1 receptor agonists or SGLT2 inhibitors are suggested for subjects with established atherosclerotic cardiovascular disease and when there is a compelling need to prevent weight gain. Furthermore, SGLT2 inhibitors are suggested for subjects with chronic kidney disease. When there is a compelling need, in other subjects, to minimize the risk of hypoglycemia, DPP-4 inhibitors, thiazolidinediones, SGLT2 inhibitors, or GLP-1 receptor agonists may be added, except when cost is a major issue, in case thiazolidinediones or sulfonylureas could be given. For DPP-4 inhibitors the position statement focused on their low risk of hypoglycemia and also that it can be given in a large group of subjects, including those with reduced renal function.

### Future Combinations

Further clinical development has focused on the potential combination between DPP-4 inhibition and SGLT2 inhibition. This combination has been suggested as a potential early glucose-lowering treatment of type 2 diabetes due to their complementing mechanism of action (142). Clinical studies have also verified augmented glucose-lowering action of this combination in association with low risk for hypoglycemia (115, 143, 144).

### Mechanism of Action

In parallel with the path to clinical development of DPP-4 inhibition, several studies have been undertaken to understand its mechanism of action. The primary pharmacology involves inhibition of the inactivation of GLP-1 and GIP, which leads to the important effects to stimulate insulin secretion and inhibit glucagon secretion in a glucose-dependent manner (145). However, there are also secondary pharmacology effects due to the diverse actions of these two hormones such that effects on autonomic nerve activity and hepatic glucose production through gut and portal GLP-1, effects on islet hormone secretion and islet inflammation through intra-islet effects, and effects through other mediators apart from GLP-1 (145–147).

### Cost

For the use of DPP-4 inhibitors, cost has been a restraining factor. In particular, in relation to thiazolidinediones and sulfonylureas, the cost is higher for DPP-4 inhibitors and therefore these two therapeutics are suggested when cost is an important issue. This will probably change when generic DPP-4 inhibitors will enter the market after the phase that the current DPP-4 inhibitors are covered by patents. This phase will most likely occur in the early years of the 2020s. How this will change the current landscape of gucose-lowering strategies will be of interest to follow.

## Conclusions and Lessons Learned

The potential of DPP-4 inhibition as a glucose-lowering concept has now been explored for more than 25 years and it is more than 10 years since several DPP-4 inhibitors were introduced to the market (Figure 1). There are several lessons learned by this development. One is that the development of DPP-4 inhibition is a rational drug design based in scientific studies on pathophysiology of type 2 diabetes and that the development shows that it is important to target both the impaired insulin secretion and the high glucagon. Another lesson which has been learned is that this development has been made possible through academic science in collaboration with the research-oriented pharmaceutical industry. A third lesson is that such a development takes time and requires focused efforts with persistence and long-term perserverance. Finally, a fourth lesson is that the development has indeed been fruitful not for only the generation of new agents to be introduced to the patients but also for the scientific field, since novel scientific discoveries have been made throughout these years in this development.

## Data Availability

No datasets were generated or analyzed for this study.

## Author Contributions

The author confirms being the sole contributor of this work and has approved it for publication.

### Conflict of Interest Statement

BA has received fees for lecturing or participating in advisory boards from Novartis, Merck, Boehringer Ingelheim, Takeda, Astra Zeneca, Novo Nordisk, Sanofi and GSK, which are companies developing and producing DPP-4 inhibitors or GLP-1 receptor agonists. BA has received research funding from Merck, Novartis and Novo Nordisk.

## Footnotes

^1^Press release Boehringer Ingelheim, February 19, 2019.

^2^Press release Eli Lilly November 5, 2018.
